# Observations on Fever and Other Diseases of the South and West

**Published:** 1829-03

**Authors:** 


WESTERN JOURNAL

OF THE

MEDICAL & PHYSICAL SCIENCES.

MARCH, 1829.

### ESSAYS AND CASES.

ART. I.—*Observations on Fever and other Diseases of the South and West.* By MEDICUS.* No. II.

Perhaps the most interesting physiological fact, which has been brought before the medical world for many years, is the elegant, because natural division of the nervous system, as taught by Mr. Charles Bell. This system or division is beautiful and interesting, not only as relates to the connection of the various parts of the apparatus with themselves, but as regards the other tissues which they control, and the organs which they regulate. What can be more philosophic in the conception, or truer in fact, than that nature should prepare and arrange one set of nerves to govern the process of digestion; another to preside over the important business of respiration; a third for the powers of voluntary motion; and, finally, a fourth to perform the exalted functions of external sensation, and connect us with each other and with all surrounding objects.

How important to the pathologist to know this; and to know,

*It is proper to state that we were mistaken in saying that this writer is a graduate of Transylvania. He was an *eleve* of that institution, but graduated east of the mountains. ED.

also, that these divisions are so connected with each other, that rudeness offered to either, will, if persisted in, provoke the energy and resistance of the whole. To illustrate this, suppose marsh poison be presented to the skin, and the mucous membranes which line the interior of the system. Between these two surfaces, lie all the vital parts of the body. Widely extended over them is that division of the nerves allotted to nutrition; and, indeed, it is internally blended and connected with all the secreting organs of the body, whether the matter, separated from the circulating mass, be excrementitious or nutrient. I would again remark, that the noxious agent *must necessarily* be first applied to the skin, or mucous membrane; for the plain reason, that they are exterior to all the other parts.

The first impression is made on the nervous tissue of these parts, because the nervous is exterior to all other organized tissues. We feel the pain arising from a prick with a thorn or a needle, before the muscles are effected or the blood made to flow. We feel nausea from an emetic, before the vasular system is deranged, or the contraction of the abdominal muscles takes place. These feelings are confessedly experienced first by the nerves adapted to that office, and conveyed to the centre of perception by that division which governs sensation.

The nerves of the skin and mucous membrane, concerned in nutrition, have a duty to perform in common with those of the other secreting organs of the system, to wit, the defence and restoration of the body; and early in the progress of disease, they cause a correspondent impression to be created throughout the division controlling nutrition, growth and decay. When the nutritive nerves are unhealthy, secretion is imperfectly performed. All the secretions require for their performance an organ, nervous energy, and blood. The organ makes, by the agency of the nervous energy, from or out of the blood, whatever secretion in the animal economy it is destined to create or produce.

That a secretory process should be properly done, the blood must possess an aptitude to be acted upon by the organ, under nervous power; but if the nerves of nutrition in the skin and mucous membranes be disordered, and if they call to their aid a portion of the nervous energy of the other organs, those organs cannot manufacture the secreted fluid as they would do in health. The consequence is, that the blood goes, unrefined and unassimilated, through the system from organ to organ, and neither of them is able to purify or prepare it. Entering the veins it is returned to the heart, and then undergoes the pulmonic circulation. The lungs, because of their inability to bear the impress of such blood, and properly vitalize it, labour and become oppressed. Unvitalized, and in a state of comparative impurity, the blood repasses to the heart, again to be dispersed to all parts of the body. In its progress it fails to give to each or any part its due share of vitality; and consequent injury is sustained by the nerves and muscles of voluntary motion.

In a state of health, if by venesection we cause a deficiency of the vitalizing principle, a corresponding deficiency takes place, in the action displayed by the nerves and muscles of voluntary motion. After the blood has been repeatedly presented to the organ of secretion, and as repeatedly passed through them unprepared; the powers of nutrition, of respiration, and of voluntary motion, are all impaired or suspended; some organ or surface, either naturally imperfect, or accidentally debilitated, yields to the enemy, becomes engorged and obstructed; a portion of the energy of each divison of the nervous system, is then directed to the support, or becomes connected with the disordered condition, of the suffering organ.

The division which presides over sensation is impaired in its power. Hence arises false vision, tinitus aurium, depraved taste, unnatural odors, and unreal pain. Thus it is that the whole nervous system becomes diseased.

In the symptoms of disease, arising from whatever cause they may, the blood vessel system is neither the first nor only one which is disordered or unbalanced. The nervous power as, remarked, is of equal importance in the sustentation of the individual: it is, in fact, co-extensive in its diffusion with the blood vessels. The symptoms which denote an unbalanced or diseased nervous action, are those which are first manifested; and from this I infer, that the nerves are the strings which first sound discordantly through the living body.

Those disorders as remarked heretofore, which partake of the nature of, or belong to the neurotic classs, are confessedly of a cold or low temperature. That is, they produce a diminution of the temperature of the body. Those individuals who are by nature possessed of a nervous temperament, have generally a coldness of the skin, and a low vascular action.—In apoplexy, hysteria, epilepsy, terror and all diseases which are produced by nervous excitement, coldness is nearly the first symptom presented. It is as invariable in its presence as any other symptom, and indeed more so. Whenever the nerves reign, coldness is the consequence.

If we reflect on the condition under which the body labours, when this tissue holds the government; and compare it with the opposite condition called vascular excitement, the conclusion follows, that though in health they harmonize very well, yet in disease they are a kind of rivals for ascendancy. It is the tendency of an exalted vascular action to increase the heat of the body, as it is that of a preponderating nervous excitement to depress it.

In the cold or nervous stage of a disorder, the action of the muscular tissue may be, and often is violent; but this action is involuntary, irregular, and agitative, producing the shake of an intermittent, and the tremor of fear, the unconnected movements of hysteria, the contortions of epilepsy, and the whimsical gesticulations of Saint Vitus' dance. On the other hand, the action of the body under vascular excitement is powerful, voluntary and determinate, and when moved, it is by the consent of the will, and for the accomplishment of some object, either really or suppositiously, within the reach of the individual.

The difference in the effects produced in the system when one or the other of the tissues has the ascendancy in action, is so palpable, as strongly to impress every clinical physician, with the fact, that each has its own peculiar mode of operating. He will also recollect, that when there is a preponderance of action in neither, and when there is no tendency to such a preponderance, the body is in a situation entirely different from either of the others, and indeed in a state of health.

The vascular system yields to the controlling action of the nerves, and the coldness of the apyrexial state depends upon this controlling action. When the vascular action is high, the nervous energy has yielded, and heat or fever is the consequence of this yielding. When they are both well balanced throughout the whole system, it is in a sound and physiological condition.

In showing how the cold fit is produced, but more particularly in treating of the order in which the nerves become impaired, I have already referred to the manner in which the blackness of the blood is produced; but here I will repeat, explicitly, that it depends upon deficient secretion.

From the skin and lungs (and mucuous membranes elsewhere) the blood is by the capillaries conveyed to the return vessels, and by them to the heart. In consequence of their inadequacy to the business of secretion, as heretofore explained, that from the skin contains what should have passed out as perspirable matter, which is emptied into the right auricle of the heart; and that from the lungs is loaded with the matter of pulmonary exhalation and conducted to the left ventricle. The coronary circulation, or the circulation of the heart, is carried on with unvitalized blood, or with blood in a state of partial impurity, so are the other two, the general circulation and the pulmonic; both of which must be supplied with this black or depraved blood. One office of the lungs is to secrete from the blood, carbon or carbonic acid, which retained, causes its dark colour; but separated and cast out, admits of the scarlet appearance which we see in arterial blood. This appearance is an evidence to us, that it is vitalized. The blackness of the blood does not depend upon marsh miasma or cold, but upon a deficient action in the secerning organs.

I have already remarked, that when the lungs fail, the three circulations, are carried on with this black or unvitalized blood. It is in its quality unnatural. The central organ of the circulation, is not only assaulted in its great cavities by this unnatural stimulus, but its very substance becomes irritated. Finding, that by its ordinary exertion it cannot rid itself of this unwelcome tenant, the black blood, it becomes excited, increases its activity, and the nervous system, fatigued by its exertion and debilitated by the remote cause, yields to the increasing action of the vascular system. The heart contracts and dilates with force and celerity. It throws the blood out upon the capillaries; they, after it has been ineffectually applied to the skin and other secretory organs to be renovated in quality, return it to the heart; thence it is cast into the lungs, where its presence irritates the respiratory nerves, respiration becomes accelerated, and the blood, black and unærated, is forced back to the heart, which is increased in the vehemence and force of its contraction. Thus is brought on the stage excitement. A stage in which the vascular completely triumphs over the nervous system. The centre of circulation has government over the centre of perception, and thus they have ascendancy, *tour a tour*, in our ordinary diseases.

Whenever the disease partakes of a general, or constitutional character, or throws into disarray the whole body, it is ushered in with a cold or chilly sensation, because the nerves always experience the first impression. In those cases of poisoning, or of severe accidents, in which death is *suddenly* produced, no congestion occurs in the venæ cavæ, or portal circle; and the blood has not the same dark or black appearance, as is observed to take place in deaths brought on more slowly. The cause is obviously this. The nervous tissue is so immediately injured, as not to lend aid to the organs of circulation. The blood may pass a few rounds, and but few. Death does not arise from "blackness of the blood and consequent weakness of the heart," but from the prostrating operation of the destructive agent on the nerves. Death occurs so soon after the accident, that the blood has not had time to be changed greatly, nor has reaction caused a congestion or engorgement. There may be a co-agulation of blood in the great vessels about the heart, but in strictly professional language there is no congestion. Nor do congestions occur during the nervous state. They arise because of increased vascular action.

In consequence of a casual or congenital proclivity to weakness, some organ yields to this strong and rapid motion of the blood. Debility arising from any cause, incapacitates an organ for the performance of its part in this accelerated circulation. Here, then, occurs "the stagnant juices which lie out of the verge of circulation," as spoken of by Langrish. I should esteem it quite an imaginary supposition, indeed, that the blood would, when impelled by an immense and powerful *vis a tergo*, pursue the common channels of circulation, and wait till quietude was restored for the opportunity to creep "out of the verge of the circulation."

If there be no physical liability to disease in any part, then, the liver is most likely to suffer. The sluggish venous circulation of that organ, its expansibility, and the soft and yielding parts by which it is surrounded, contribute to make it the seat of obstruction or the receptacle of blood.

The temporary loss of so much blood out of the general circulation, diminishes the quantity of impure blood returned to the heart. Another circumstance also contributes to tranquilize that organ. It is this. By contracting with increased frequency and force, it has thrown the blood an increased number of times upon the skin and lungs; which, though they vitalize it imperfectly, at each time, yet in the end do much for the benefit of that fluid, the heart becomes tranquil, and immediately the preparation for the cold stage, or the nervous action commences. It is brought about, is carried on and terminated in the manner before explained.

In writing on the pathology and treatment of the diseases of the south and west, I shall have ample opportunities of illustrating this subject.

(TO BE CONTINUED.)

